# Supplementary material for: Genome-wide identification of metabolic and regulatory determinants of intracellular growth in Brucella neotomae
Source: bioRxiv. 2026 Apr 6:2026.04.05.716554. Preprint. [Version 1] doi: 10.64898/2026.04.05.716554 (PMC13081920; doi:10.64898/2026.04.05.716554)
Supplement: Supplement 5 — Fig. S5. Intracellular growth of histidine biosynthesis mutants. [file media-5.pdf]

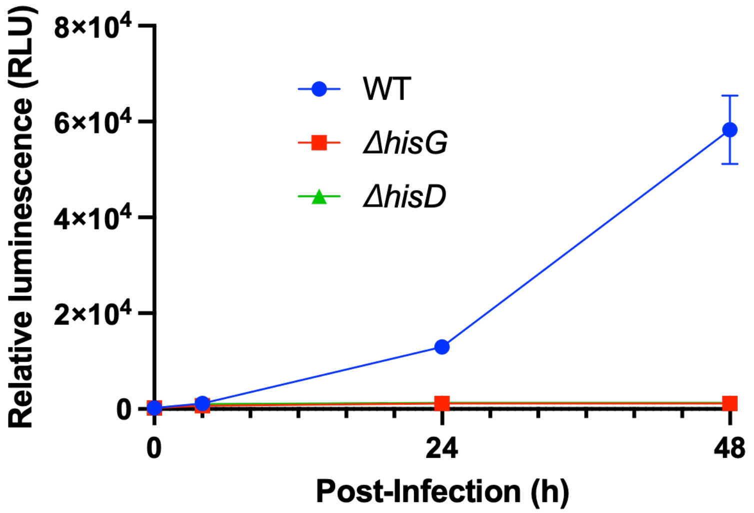

S5 Figure. Intracellular growth of *B. neotomae* histidine biosynthesis mutants. Intracellular growth of the wild-type (WT) strain and the Δ*hisG* and Δ*hisD* mutant strains was monitored at 0, 4, 24, and 48 hours post-infection. J774A.1 macrophages were infected at a multiplicity of infection (MOI) of 10. Intracellular bacterial replication was quantified by measuring luminescence emitted from a chromosomally integrated *lux* operon. Data represent the mean of four independent experiments, with standard deviations indicated.
