# Supplementary material for: Genome-wide identification of metabolic and regulatory determinants of intracellular growth in Brucella neotomae
Source: bioRxiv. 2026 Apr 6:2026.04.05.716554. Preprint. [Version 1] doi: 10.64898/2026.04.05.716554 (PMC13081920; doi:10.64898/2026.04.05.716554)
Supplement: Supplement 13 — Table S13. Bacterial strains, plasmids, cell lines, and PCR primers used in this study. Comprehensive list of strains, plasmids, expression constructs, and PCR primers used for mutant construction, complementation, promoter analysis, and gene expression studies. [file media-13.pdf]

Table S13. Bacterial strains, plasmids, cell lines, and PCR primers used in this study.

**Bacterial strains**		
Strain	*Relevant characteristics*	*Source or Reference*
B. neotomae 5K33	Parent biosafety level 2 rodent pathogen	BEI Resources
B. neotomae-Lux	Transposon mutant of 5K33 expressing Lux operon-nat genes	[1]
B. neotomae-tdTomato	Transposon mutant of 5K33 having proD/tdTomato-nat genes	
B. neotomae ΔvirB4	virB4 in-frame deletion mutant of B. neotomae 5K33	
Escherichia coli NEB-5α	*fhuA2 Δ(argF-lacZ)U169 phoA glnV44 Φ80Δ(lacZ)M15 gyrA96 recA1 relA1 endA1 thi-1 hsdR17*	NEB
E. coli Ec100D pir-116	*F- mcrA Δ(mrr-hsdRMS-mcrBC) ϕ80dlacZΔM15 ΔlacX74 recA1 endA1 araD139 Δ(ara, leu)7697 galU galK λ- rpsL nupG pir-116(DHRF)*	Bioresearch Technologies
E. coli β2155	SM10 λpir derivative; *ΔdapA::erm* DAP auxotroph donor strain for conjugation	[2]
**Plasmids**		
pMAR2xT7	Amp^R, Himar1 transposase	[3]
pSR47s	R6K, sacB, Km^R, suicide vector	[4]
pBMTL2	Km^R, broad host range vector	[5]
pBMTL3	Cam^R, broad host range vector	[5]
pBAD	Amp^R, protein expression vector	Addgene (#37129)
**Cell Lines**		
Cell Lines	*Relevant characteristics*	*Source*
J774A.1	Mouse macrophage cell line, ATCC TIB-67	ATCC
**PCR primers**		
Name	*Sequences*	*Characteristics*
NatpMar2X-F	CCCGGTCTGGAGGACAGTAATGGGTACGACCCTTGATGACA	Amplification of NAT gene for pMAR2X
NatpMar2x-R	GTTGGCTGATAAGTCCCCGGTCTTTACGGGCAGGGCATGCTCATG	
pMAR2xNat-F	GGGGACTTATCAGCCAACCTGTTCCGACAGGGCCCAATTCGCCCTATAG	Amplification of pMAR2X for NAT gene
pMAR2xNat-R	TACTGTCCTCCAGACCGGGGACTTATCAGCCAACCTGTTCCGACA	

trpD-1F	CGC *GGA TCC* AAG GTG AAC CAG GCC GTC	*trpD* in-frame mutant
trpD-1R	CCC ATC CAC TAA ACT TAA ACA ACC GGC GAT TTC CGG CAC	
trpD-2F	TGT TTA AGT TTA GTG GAT GGG CTG CTC AAT TCA GGC GCC	
trpD-2R	CGC *GAG CTC* AGG CTT CGA TCT TGC GAA	
trpDc-F	CGC *TCT AGA* ATGGCTGATTTGAAACCC	*trpD* complementation
trpDc-R	CGC *GAT ATC* TCAGGCCGGCTTGTCGTT	
metH-1F	CGC *GGA TCC* GGC ATT ACA ACC AGA CAA	*metH* in-frame mutant
metH-1R	CCC ATC CAC TAA ACT TAA ACA GGT CAA GGT CAG AAG ATC	
metH-2F	TGT TTA AGT TTA GTG GAT GGG GTG TCG GGT CTC TAT ATC	
metH-2R	CGC *GAG CTC* GCT GTT TTG CCG TTT CCT	
metHc-F	CGC *TCT AGA* ATGGCGTCTTCCCTTGAC	*metH* complementation
metHc-R	CGC *GAT ATC* TCAGGCCGCTTCGTCTTT	
qmetH-F	GCCAGCTTCAGGGCAATA	*metH* qPCR
qmetH-R	CGATGGAAGTGGAGGAGAAAG	
hisG-1F	CGC *GGA TCC* GACCATGCTTGGCGCTTG	*hisG* in-frame mutant
hisG-1R	CCC ATC CAC TAA ACT TAA ACA CCTCGCCTTCGACGCGCG	
hisG-2F	TGT TTA AGT TTA GTG GAT GGG ATCACCTCAACCGGCTCCAC	
hisG-2R	CGC *GAG CTC* GCTGAACCGTCGGCCACT	
hisGc-F	CGC *TCT AGA* ATGAGCGTCACATTGGCA	*hisG* complementation
hisGc-R	CGC *GAT ATC* TTATATTTCCAGTCCCGC	
qhisG-F	CGGATGACGACCGCAATTA	*hisG* qPCR
qhisG-R	AGATCGACGCTGCCATAAC	
hisD-1F	CGC *GGA TCC* CGTATGAGAACGCCCTCC	*hisD* in-frame mutant
hisD-1R	CCC ATC CAC TAA ACT TAA ACA CTCGCGGCGTACACGATCCA	
hisD-2F	TGT TTA AGT TTA GTG GAT GGG CGTTTCTCCTCCGGCCTT	
hisD-2R	CGC *GAG CTC* CGGGCATTGGGAACGTCG	
virB4q-F	GGCACTGAAACCATCGAGATAC	*virB4* qPCR
virB4q-R	GCTATACATCAGGCCGTTCAAA	
PvirB1-F	TGCCGCCTTGTTCACCGG	*virB1* promoter region
PvirB1-R	AGGATCGTCTCCTTCTCA	
rsh-1F	CGC *GGA TCC* CGGTTCGAGGGAGTTTCG	*rsh* in-frame mutant
rsh-1R	CCC ATC CAC TAA ACT TAA ACA GATTGTCGCCTCGTCCAA	
rsh-2F	TGT TTA AGT TTA GTG GAT GGG TTGGCGGAAATTGCCCAG	
rsh-2R	CGC *GAG CTC* AGGCCGGAGGTGAGGATA	
rshc-F	CGC *TCT AGA* ATGATGCGCCAATATGAG	*rsh* complementation
rshc-R	CGC *GAT ATC* CTATCCGTTCACACGCTT	
rshq-F	TGCGCGTTCTTCTGGTAAA	*rsh* qPCR
rshq-R	CATGGTCTCCTCGGCAATAC	

deoR-1F	CGC *GGA TCC* GCA AAA CGG AAA GCG GCC	deoR/*glpR* in-frame mutant
deoR-1R	CCC ATC CAC TAA ACT TAA ACA CTG TTC TTC CAG CGA ATG	
deoR-2F	TGT TTA AGT TTA GTG GAT GGG GTC GTT ACC GAT GCG GGC	
deoR-2R	CGC *GAG CTC* GAT CAA ACG ACT GCC CAG	
lysR-1F	CGC *GGA TCC* ATTCATTCCATCGCATAA	*lysR*-family in-frame mutant
lysR-1R	CCC ATC CAC TAA ACT TAA ACA AAGATTGCGAATGGCGGA	
lysR-2F	TGT TTA AGT TTA GTG GAT GGG CGCCAGTTTCTCATGCTG	
lysR-2R	CGC *GAG CTC* GAATATAATTTATATTGTGG	
bvrR-1F	CGC*GGATCC*AGATTTGAACCGGTACAT	*bvrR* in-frame mutant
bvrR-1R	CCC ATC CAC TAA ACT TAA ACA CGGCATCTTGATATCGAA	
bvrR-2F	TGT TTA AGT TTA GTG GAT GGG GTGGATGATCGCACCATC	
bvrR-2R	CGC*GAGCTC*ATTGCTCAACGCGCCGTC	
PbvrR-1F	GGAAATCGAAGCGGCCTT	*bvrR* promoter region
PbvrR-2F	GGTGTGGAAAACCGCAAA	
PbvrR-R	GAGGTCAGGATGTTGCGG	
NHBvrR-F	CGC *AGATCT* AAGGAAGCTTCGGCAACG	*bvrR*_N'-His expression
NHBvrR-R	CGC *GAATTC* TTACGCTTCCCGGAAACG	
CHBvrR-F	CGC *CCATGG* ATGAAGGAAGCTTCGGCA	*bvrR*_C'-His expression
CHBvrR-R	CGC *GAATTC* TCAGTGGTGGTGGTGGTGGTGCGCTTCCCGGAAACGATAA	
pBAD33-F	GGAAGCGTAAGTCGACCTGCAGGCATGCAA	pBAD33 for *bvrR* conditional knockout
pBAD33-R	GGTTAATTCCTCCTGTTAGCTAGCCCAAAAAAACGGGT	
bvrR-F	TAACAGGAGGAATTAACCATGAAGGAAGCTTCGGCAAC	*bvrR* conditional knockout
bvrR-R	GCAGGTCGACTTACGCTTCCCGGAAACGAT	
bvrS-F	CGC *AGATCT* GTCGCAGAGACCCAGAAA	*bvrS* expression
bvrS-R	CGC *TTCGAA* TCATGCGCTGGCCGGAAG	
bvrRc-F	CGC *TCT AGA* ATGAAGGAAGCTTCGGCA	bvrR complementation
bvrRc-R	CGC *GAT ATC* TTACGCTTCCCGGAAACG	
ompR1-1F	CGC *GGA TCC* CAG CCA TAA TCT GGG ATG	*ompR1* in-frame mutant
ompR1-1R	CCC ATC CAC TAA ACT TAA ACA GCC AGC CAT CGT GAC GCG	
ompR1-2F	TGT TTA AGT TTA GTG GAT GGG CCC GCA AAC CCG GTC TGG	
ompR1-2R	CGC *GAG CTC* GCT TTC CAT GAA CCA CAT	
ompR1-F	CGC *AGATCT* GCTGCAGAAGAAGAAAAG	*ompR1* expression
ompR1-R	CGC *GAATTC* CTATTCGATGCTGAGTTT	
ompR1c-F	CGC *TCT AGA* ATGGCTGCAGAAGAAGAA	ompR1 complementation
ompR1c-R	CGC *GAT ATC* CTATTCGATGCTGAGTTT	
PompR1-F	CATGGTCAATCCCGAGCG	*ompR1* promoter
PompR1-R	CGAAACCCGCTATTGGTC	
PEnvZ1-F	CGC *AGATCT* AGATCGCCCTTCAGAAGA	

PEnvZ1-R	CGC *GAATTC* TCAAGCCGGAATATGAAT	*envZ1* expression
rpoN-1F	CGC *GGA TCC* CCGGTTCCCACTTTTGGG	*rpoN* in-frame mutant
rpoN-1R	CCC ATC CAC TAA ACT TAA ACA CGTCATCTGTAGCAGCTTGA	
rpoN-2F	TGT TTA AGT TTA GTG GAT GGG GCCATCGTGGATGCGCTG	
rpoN-2R	CGC *GAG CTC* GGCTTGATGATTTCGGCT	
vjbR-1F	CGC *GGA TCC* GATCTCGTTCATTTTCCG	*vjbR* in-frame mutant
vjbR-1R	CCC ATC CAC TAA ACT TAA ACA CTGAAAAACCGGGTCAAT	
vjbR-2F	TGT TTA AGT TTA GTG GAT GGG GAAATCGCCGAAATCCTC	
vjbR-2R	CGC *GAG CTC* GAGCTTTTCTTTTCGCCT	
vjbR-F	CGC *AGATCT* GCGCTTCTAACCCGCATCCGG	*vjbR* expression
vjbR-R	CGC *GAATTC* TCAGACGAGATGCTGTACCTC	
PvjbR-F	CTTCGGTGCGCTTGCGGA	*vjbR* promoter region
PvjbR-R	AGTATCGCTTTGAAAGGA	
vjbRc-F	CGC *TCT AGA* ATGGCGCTTCTAACCCGC	vjbR complementation
vjbRc-R	CGC *GAT ATC* TCAGACGAGATGCTGTAC	
aqpZ-1F	CGC GGA TCC GAT TCG CAT ACT TGC CGT	aqpZ in-frame mutant
aqpZ-1R	CCC ATC CAC TAA ACT TAA ACA GGT GAG GAC GGT TAA ACC	
aqpZ-2F	TGT TTA AGT TTA GTG GAT GGG CGC TCG ACC GGC GTT GCC	
aqpZ-2R	CGC GAG CTC TAT TCG AAT CCG GCC CAT	
aqpZc-F	CGC *TCT AGA* ATGTTGAACAAATTATCG	aqpZ complementation
aqpZc-R	CGC *GAT ATC* TTAATCTCGGCCGAGCAG	
NLuc-F	CGC *TCTAGA* TAA GGA GGA AAA AAA ATGGTCTTCACACTCGAA	pBMTL3-NLuc
NLuc-R	CGC *AAGCTT* TTACGCCAGAATGCGTTC	
NLucPvirB1-F	CGC *GGATCC* TGCCGCCTTGTTCACCGG	pBMTL3-PvirB1_NLuc
NLucPvirB1-R	CGC *TCTAGA* AGGATCGTCTCCTTCTCA	
NLucPbvrR-F	CGC *GGATCC* GGAAATCGAAGCGGCCTT	pBMTL3-PbvrR_NLuc
NLucPbvrR-R	CGC *TCTAGA* GGTGTGGAAAACCGCAAA	
qrpoB-F	TCAGCGCGATCTGACTTATTC	*rpoB* qPCR
qrpoB-R	CTGCTCCTTGATGTCCTTGAT	
qompR-F	GCAGCCTTCTCTCACAATATCT	*ompR* qPCR
qompR-R	CGAGAATCAGAAGGTCGAAGTC	
qbvrR-F	TTATCGCGTCGAAACCTATACC	*bvrR* qPCR
qbvrR-R	ATGCGCGGCATCTTGATA	
qvjbR-F	AGCCGATCTGACTGTTCTTATG	*vjbR* qPCR
qvjbR-R	GTAATACGGAGCGTCTTCCTG	

1. The underlined italicized sequence indicates the restriction enzyme recognition site.

References

1. Kang YS, Kirby JE. Promotion and Rescue of Intracellular Brucella neotomae Replication during Coinfection with Legionella pneumophila. Infect Immun. 2017;85(5):e00991-16. Epub 2017/04/21. doi: 10.1128/IAI.00991-16. PubMed PMID: 28264909; PubMed Central PMCID: PMCPMC5400850.
2. Dehio C, Meyer M. Maintenance of broad-host-range incompatibility group P and group Q plasmids and transposition of Tn5 in Bartonella henselae following conjugal plasmid transfer from Escherichia coli. J Bacteriol. 1997;179(2):538-40. doi: 10.1128/jb.179.2.538-540.1997. PubMed PMID: 8990308; PubMed Central PMCID: PMCPMC178726.
3. Liberati NT, Urbach JM, Miyata S, Lee DG, Drenkard E, Wu G, et al. An ordered, nonredundant library of Pseudomonas aeruginosa strain PA14 transposon insertion mutants. Proc Natl Acad Sci U S A. 2006;103(8):2833-8. Epub 20060213. doi: 10.1073/pnas.0511100103. PubMed PMID: 16477005; PubMed Central PMCID: PMCPMC1413827.
4. Andrews HL, Vogel JP, Isberg RR. Identification of linked Legionella pneumophila genes essential for intracellular growth and evasion of the endocytic pathway. Infect Immun. 1998;66(3):950-8. Epub 1998/03/06. doi: 10.1128/iai.66.3.950-958.1998. PubMed PMID: 9488381; PubMed Central PMCID: PMCPMC108001.
5. Lynch MD, Gill RT. Broad host range vectors for stable genomic library construction. Biotechnol Bioeng. 2006;94(1):151-8. Epub 2006/02/24. doi: 10.1002/bit.20836. PubMed PMID: 16496398.
